# 2-(4-Isobutyl­phen­yl)-*N*′-[1-(4-nitro­phen­yl)ethyl­idene]propanohydrazide

**DOI:** 10.1107/S1600536809003420

**Published:** 2009-02-04

**Authors:** Hoong-Kun Fun, Suchada Chantrapromma, K. V. Sujith, B. Kalluraya

**Affiliations:** a X-ray Crystallography Unit, School of Physics, Universiti Sains Malaysia, 11800 USM, Penang, Malaysia; b Crystal Materials Research Unit, Department of Chemistry, Faculty of Science, Prince of Songkla University, Hat-Yai, Songkhla 90112, Thailand; c Department of Studies in Chemistry, Mangalore University, Mangalagangotri, Mangalore 574 199, India

## Abstract

The mol­ecule of the title compound, C_21_H_25_N_3_O_3_, exists in a *trans* configuration with respect to the ethyl­idene unit. The dihedral angle between the two substituted benzene rings is 86.99 (7)°. The nitro group is twisted from the attached benzene ring at an angle of 17.02 (7)°. In the crystal structure, mol­ecules are linked by pairs of N—H⋯O hydrogen bonds in a face-to-face manner into centrosymmetric dimers. These dimer units are further linked into chains along the *c* axis by weak C—H⋯O inter­actions. These chains are stacked along the *b* axis. The crystal is further stabilized by weak C—H⋯π inter­actions.

## Related literature

For reference structural data, see: Allen *et al.* (1987 ▶). For related structures, see, for example: Fun *et al.* (2008 ▶). For background to the activities and applications of hydrazones, see, for example: Amir & Kumar (2007 ▶); Bedia *et al.* (2006 ▶); Pasha & Nanjundaswamy (2004 ▶); Rollas *et al.* (2002 ▶); Sridhar & Perumal (2003 ▶); Terzioglu & Gürsoy (2003 ▶).
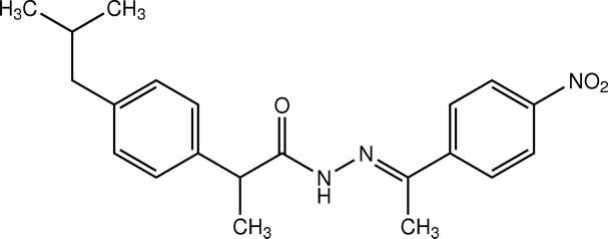


## Experimental

### 

#### Crystal data

C_21_H_25_N_3_O_3_
*M* *_r_* = 367.44
Monoclinic, 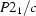

*a* = 13.7343 (2) Å
*b* = 7.9039 (2) Å
*c* = 20.8408 (3) Å
β = 122.677 (1)°
*V* = 1904.29 (7) Å^3^
*Z* = 4
Mo *K*α radiation
μ = 0.09 mm^−1^
*T* = 100.0 (1) K
0.58 × 0.20 × 0.10 mm

#### Data collection

Bruker SMART APEX2 CCD area-detector diffractometer
Absorption correction: multi-scan (**SADABS**; Bruker, 2005 ▶) *T*_min_ = 0.952, *T*_max_ = 0.991
24946 measured reflections
5506 independent reflections
4402 reflections with *I* > 2σ(*I*)
*R*_int_ = 0.041

#### Refinement

*R*[*F* ^2^ > 2σ(*F* ^2^)] = 0.046
*wR*(*F* ^2^) = 0.122
*S* = 1.05
5506 reflections
248 parameters
H-atom parameters constrained
Δρ_max_ = 0.37 e Å^−3^
Δρ_min_ = −0.28 e Å^−3^

### 

Data collection: *APEX2* (Bruker, 2005 ▶); cell refinement: *APEX2*; data reduction: *SAINT* (Bruker, 2005 ▶); program(s) used to solve structure: *SHELXTL* (Sheldrick, 2008 ▶); program(s) used to refine structure: *SHELXTL*; molecular graphics: *SHELXTL*; software used to prepare material for publication: *SHELXTL* and *PLATON* (Spek, 2003 ▶).

## Supplementary Material

Crystal structure: contains datablocks global, I. DOI: 10.1107/S1600536809003420/is2386sup1.cif

Structure factors: contains datablocks I. DOI: 10.1107/S1600536809003420/is2386Isup2.hkl

Additional supplementary materials:  crystallographic information; 3D view; checkCIF report

## Figures and Tables

**Table 1:** Hydrogen-bond geometry (Å, °) *Cg*1 and *Cg*2 are the centroids of the C1–C6 and C10–C15 rings, respectively.

*D*—H⋯*A*	*D*—H	H⋯*A*	*D*⋯*A*	*D*—H⋯*A*
N1—H1⋯O1^i^	0.89	2.13	3.0012 (16)	167
C1—H1*A*⋯O2^ii^	0.93	2.58	3.4118 (15)	149
C16—H16*B*⋯*Cg*1^iii^	0.97	2.88	3.6113 (16)	133
C18—H18*B*⋯*Cg*2^iv^	0.96	2.99	3.9348 (16)	167
C21—H21*C*⋯*Cg*1^v^	0.96	2.80	3.5600 (16)	137

Symmetry codes: (i) 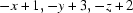; (ii) 
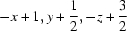
; (iii) 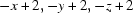; (iv) 
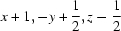
; (v) 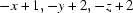.

**Fig. 1.**
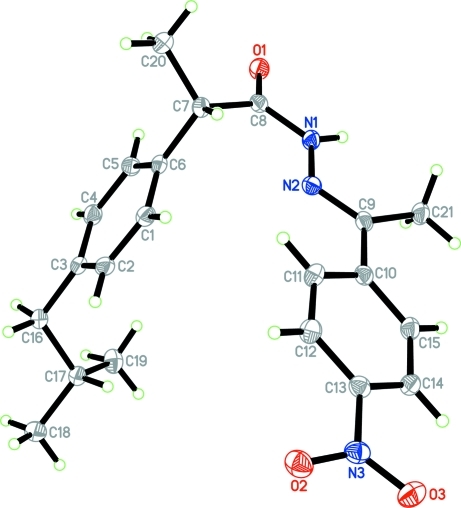
The structure of (I), showing 50% probability displacement ellipsoids and the atom-numbering scheme.

**Fig. 2.**
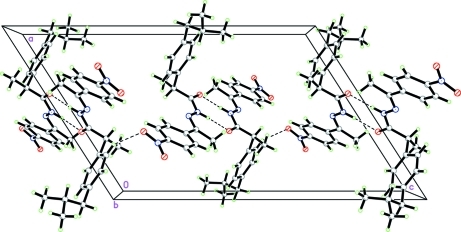
The packing diagram of (I), viewed along the b axis, showing dimers linked by C—H···O interactions into a chain along the c axis. Hydrogen bonds and weak interactions are shown as dashed lines.

## supplementary crystallographic information

### Comment

Hydrazones have been prepared by treating aryl hydrazines with carbonyl compounds using a variety of solvents in presence or absence of an acidic catalyst. (Pasha & Nanjundaswamy, 2004). Aryl hydrazones are important building blocks for the synthesis of a variety of heterocyclic compounds such as pyrazolines and pyrazoles (Sridhar & Perumal, 2003). Hydrazones have been demonstrated to possess variety of pharmacological activities (Bedia *et al.*, 2006; Rollas *et al.*, 2002; Terzioglu & Gürsoy, 2003). These observations have been the guidelines for the development of new hydrazones that possess varied biological activities. Similarly ibuprofen is also known for their pharmaceutical activities and belongs to the class of non-steroidal anti-inflammatory drugs (Amir & Kumar, 2007). According to our previous work, we are interested in the synthesis and crystal structure of ibuprofen containing hydrazone derivatives (Fun *et al.*, 2008). Prompted by the biological activities of hydrazones and ibuprofen, the title compound was synthesized and it crystal structure was reported here.

In the structure of the title compound, (I), the molecule exist in a *trans* configuration with respect to the ethylidene C9═N2 unit (Fig. 1). The dihedral angle between the two substituted benzene rings is 86.99 (7)°. In the 4-nitrophenyl unit, the nitro group is twisted from the mean plane of the C10–C15 ring which can be shown by the dihedral angle between the mean planes through the C13/N3/O2/O3 group and the C10–C15 ring being 17.02 (7)°. Atoms O1, N1, N2, C7, C8, C9 and C21 lie on the same plane with a maximum deviation of -0.032 (1)Å for atom N1. This plane makes dihedral angles of 73.01 (6) and 15.02 (5)° with the C1–C6 and C10–C15 benzene rings, respectively. The isobutyl substituent (C16–C19) is (-)-synclinal with respect to the C1–C6 ring with the torsion angle C2—C3—C16—C17 being -76.91 (14)°. The bond distances have normal values (Allen *et al.*, 1987) and are comparable with the related structure (Fun *et al.*, 2008).

In the crystal packing, N—H···O hydrogen bonds (Table 1 and Fig. 2) link the molecules into face-to-face dimers. These dimers are further linked into chains along the *c* axis and these chains are stacked along the *b* axis. The crystal is stabilized by N—H···O hydrogen bonding, and weak C—H···O and C—H···π interactions (Table 1); *Cg*1 and *Cg*2 are the centroids of the C1–C6 and C10–C15 rings, respectively (Table 1).

### Experimental

The title compound was obtained by refluxing 2-[4-(2-methylpropyl)phenyl]propanehydrazide (0.01 mol) and 4-nitroacetophenone (0.01 mol) in ethanol (30 ml) by adding 3 drops of concentrated Sulfuric acid for 1 h. Excess ethanol was removed from the reaction mixture under reduced pressure. The solid product obtained was filtered, washed with ethanol and dried. Colorless single crystals suitable for X-ray analysis were obtained from ethanol by slow evaporation (yield 74%; m.p. 443 K).

### Refinement

All H atoms were placed in calculated positions with d(N—H) = 0.89 Å, *U*_iso_(H) = 1.2*U*_eq_(N) for NH, d(C—H) = 0.93 Å, *U*_iso_(H) = 1.2*U*_eq_(C) for aromatic and CH, 0.97 Å, *U*_iso_(H) = 1.2*U*_eq_(C) for CH_2_, 0.96 Å, *U*_iso_(H) = 1.5*U*_eq_(C) for CH_3_ atoms. The rotating group model was used for the methyl groups. The highest residual electron density peak is located at 0.65 Å from C10 and the deepest hole is located at 1.29 Å from C9.

### Crystal data

**Table:**

C_21_H_25_N_3_O_3_	*F*(000) = 784
*M**_r_* = 367.44	*D*_x_ = 1.282 Mg m^−^^3^
Monoclinic, *P*2_1_/*c*	Melting point: 443 K
Hall symbol: -P 2ybc	Mo *K*α radiation, λ = 0.71073 Å
*a* = 13.7343 (2) Å	Cell parameters from 5560 reflections
*b* = 7.9039 (2) Å	θ = 2.0–30.0°
*c* = 20.8408 (3) Å	µ = 0.09 mm^−^^1^
β = 122.677 (1)°	*T* = 100 K
*V* = 1904.29 (7) Å^3^	Block, colorless
*Z* = 4	0.58 × 0.20 × 0.10 mm

### Data collection

**Table:**

Bruker SMART APEX2 CCD area-detector diffractometer	5506 independent reflections
Radiation source: fine-focus sealed tube	4402 reflections with *I* > 2σ(*I*)
graphite	*R*_int_ = 0.041
Detector resolution: 8.33 pixels mm^-1^	θ_max_ = 30.0°, θ_min_ = 2.0°
ω scans	*h* = −19→19
Absorption correction: multi-scan (*SADABS*; Bruker, 2005)	*k* = −10→11
*T*_min_ = 0.952, *T*_max_ = 0.991	*l* = −29→29
24946 measured reflections	

### Refinement

**Table:**

Refinement on *F*^2^	Primary atom site location: structure-invariant direct methods
Least-squares matrix: full	Secondary atom site location: difference Fourier map
*R*[*F*^2^ > 2σ(*F*^2^)] = 0.046	Hydrogen site location: inferred from neighbouring sites
*wR*(*F*^2^) = 0.122	H-atom parameters constrained
*S* = 1.05	*w* = 1/[σ^2^(*F*_o_^2^) + (0.0567*P*)^2^ + 0.5875*P*] where *P* = (*F*_o_^2^ + 2*F*_c_^2^)/3
5506 reflections	(Δ/σ)_max_ = 0.001
248 parameters	Δρ_max_ = 0.37 e Å^−^^3^
0 restraints	Δρ_min_ = −0.28 e Å^−^^3^

### Special details

**Table:**

Experimental. The low-temperature data was collected with the Oxford Cyrosystem Cobra low-temperature attachment.
Geometry. All e.s.d.'s (except the e.s.d. in the dihedral angle between two l.s. planes) are estimated using the full covariance matrix. The cell e.s.d.'s are taken into account individually in the estimation of e.s.d.'s in distances, angles and torsion angles; correlations between e.s.d.'s in cell parameters are only used when they are defined by crystal symmetry. An approximate (isotropic) treatment of cell e.s.d.'s is used for estimating e.s.d.'s involving l.s. planes.
Refinement. Refinement of *F*^2^ against ALL reflections. The weighted *R*-factor *wR* and goodness of fit *S* are based on *F*^2^, conventional *R*-factors *R* are based on *F*, with *F* set to zero for negative *F*^2^. The threshold expression of *F*^2^ > σ(*F*^2^) is used only for calculating *R*-factors(gt) *etc*. and is not relevant to the choice of reflections for refinement. *R*-factors based on *F*^2^ are statistically about twice as large as those based on *F*, and *R*- factors based on ALL data will be even larger.

### Fractional atomic coordinates and isotropic or equivalent isotropic displacement parameters (Å^2^)

**Table:**

	*x*	*y*	*z*	*U*_iso_*/*U*_eq_	
O1	0.61564 (7)	1.53156 (12)	0.98872 (5)	0.02087 (19)	
O2	0.39321 (8)	0.36757 (13)	0.73982 (5)	0.0250 (2)	
O3	0.25151 (8)	0.31704 (13)	0.75538 (6)	0.0285 (2)	
N1	0.50374 (8)	1.30105 (14)	0.94275 (6)	0.0169 (2)	
H1	0.4687	1.3350	0.9661	0.020*	
N2	0.47995 (8)	1.14801 (14)	0.90567 (5)	0.0162 (2)	
N3	0.32794 (9)	0.40891 (14)	0.76090 (6)	0.0200 (2)	
C1	0.74608 (10)	1.05444 (16)	0.93547 (7)	0.0173 (2)	
H1A	0.6838	1.0228	0.8877	0.021*	
C2	0.83450 (10)	0.93944 (16)	0.97821 (7)	0.0184 (2)	
H2A	0.8310	0.8328	0.9582	0.022*	
C3	0.92861 (10)	0.98084 (16)	1.05069 (7)	0.0176 (2)	
C4	0.93176 (10)	1.14328 (17)	1.07810 (7)	0.0195 (2)	
H4A	0.9937	1.1745	1.1260	0.023*	
C5	0.84359 (10)	1.25954 (17)	1.03485 (7)	0.0180 (2)	
H5A	0.8479	1.3672	1.0542	0.022*	
C6	0.74891 (10)	1.21660 (16)	0.96282 (6)	0.0158 (2)	
C7	0.65444 (10)	1.34418 (16)	0.91325 (7)	0.0168 (2)	
H7A	0.5983	1.2892	0.8649	0.020*	
C8	0.59144 (10)	1.40054 (16)	0.95135 (6)	0.0162 (2)	
C9	0.39775 (9)	1.05845 (16)	0.90156 (6)	0.0159 (2)	
C10	0.37649 (10)	0.89227 (16)	0.86281 (6)	0.0158 (2)	
C11	0.42259 (10)	0.85604 (17)	0.81818 (7)	0.0188 (2)	
H11A	0.4638	0.9391	0.8110	0.023*	
C12	0.40751 (10)	0.69891 (17)	0.78498 (7)	0.0193 (2)	
H12A	0.4395	0.6750	0.7564	0.023*	
C13	0.34376 (10)	0.57704 (16)	0.79498 (6)	0.0173 (2)	
C14	0.29521 (10)	0.60950 (17)	0.83742 (7)	0.0187 (2)	
H14A	0.2521	0.5272	0.8431	0.022*	
C15	0.31236 (10)	0.76689 (17)	0.87100 (7)	0.0185 (2)	
H15A	0.2804	0.7897	0.8997	0.022*	
C16	1.02083 (10)	0.85055 (17)	1.09705 (7)	0.0202 (2)	
H16A	1.0898	0.9078	1.1368	0.024*	
H16B	1.0405	0.7935	1.0643	0.024*	
C17	0.98418 (10)	0.71672 (17)	1.13418 (7)	0.0184 (2)	
H17A	0.9073	0.6748	1.0951	0.022*	
C18	1.06782 (11)	0.56769 (18)	1.16314 (8)	0.0241 (3)	
H18A	1.0453	0.4871	1.1873	0.036*	
H18B	1.1447	0.6073	1.1993	0.036*	
H18C	1.0661	0.5148	1.1211	0.036*	
C19	0.97652 (11)	0.7941 (2)	1.19870 (7)	0.0249 (3)	
H19A	0.9454	0.7121	1.2169	0.037*	
H19B	0.9270	0.8916	1.1800	0.037*	
H19C	1.0524	0.8271	1.2397	0.037*	
C20	0.70381 (11)	1.49872 (18)	0.89582 (8)	0.0226 (3)	
H20A	0.7384	1.4634	0.8685	0.034*	
H20B	0.7613	1.5519	0.9427	0.034*	
H20C	0.6427	1.5776	0.8654	0.034*	
C21	0.32900 (10)	1.11330 (18)	0.93465 (7)	0.0203 (3)	
H21A	0.2933	1.2206	0.9134	0.030*	
H21B	0.3794	1.1236	0.9890	0.030*	
H21C	0.2703	1.0307	0.9227	0.030*	

### Atomic displacement parameters (Å^2^)

**Table:**

	*U*^11^	*U*^22^	*U*^33^	*U*^12^	*U*^13^	*U*^23^
O1	0.0209 (4)	0.0185 (5)	0.0261 (4)	−0.0017 (4)	0.0145 (4)	−0.0043 (4)
O2	0.0296 (5)	0.0246 (5)	0.0256 (4)	0.0039 (4)	0.0180 (4)	−0.0002 (4)
O3	0.0281 (5)	0.0212 (5)	0.0368 (5)	−0.0066 (4)	0.0178 (4)	−0.0043 (4)
N1	0.0166 (4)	0.0165 (5)	0.0205 (5)	0.0000 (4)	0.0119 (4)	−0.0017 (4)
N2	0.0159 (4)	0.0148 (5)	0.0172 (4)	0.0005 (4)	0.0085 (4)	0.0000 (4)
N3	0.0211 (5)	0.0181 (5)	0.0180 (5)	0.0016 (4)	0.0088 (4)	0.0020 (4)
C1	0.0178 (5)	0.0173 (6)	0.0187 (5)	−0.0028 (5)	0.0110 (4)	−0.0025 (5)
C2	0.0221 (6)	0.0140 (6)	0.0243 (6)	−0.0011 (5)	0.0159 (5)	−0.0013 (5)
C3	0.0171 (5)	0.0164 (6)	0.0240 (5)	−0.0009 (5)	0.0142 (5)	0.0017 (5)
C4	0.0164 (5)	0.0205 (7)	0.0209 (5)	−0.0024 (5)	0.0097 (5)	−0.0013 (5)
C5	0.0183 (5)	0.0157 (6)	0.0216 (5)	−0.0011 (5)	0.0117 (5)	−0.0023 (5)
C6	0.0152 (5)	0.0164 (6)	0.0196 (5)	−0.0007 (5)	0.0119 (4)	0.0003 (5)
C7	0.0165 (5)	0.0179 (6)	0.0182 (5)	0.0005 (5)	0.0109 (4)	0.0002 (5)
C8	0.0144 (5)	0.0172 (6)	0.0161 (5)	0.0022 (5)	0.0076 (4)	0.0021 (5)
C9	0.0132 (5)	0.0187 (6)	0.0151 (5)	0.0025 (5)	0.0071 (4)	0.0022 (4)
C10	0.0132 (5)	0.0177 (6)	0.0150 (5)	0.0016 (5)	0.0066 (4)	0.0018 (4)
C11	0.0185 (5)	0.0210 (6)	0.0197 (5)	−0.0026 (5)	0.0122 (5)	0.0003 (5)
C12	0.0194 (5)	0.0223 (6)	0.0180 (5)	−0.0009 (5)	0.0112 (5)	−0.0008 (5)
C13	0.0159 (5)	0.0172 (6)	0.0158 (5)	0.0011 (5)	0.0066 (4)	0.0001 (5)
C14	0.0168 (5)	0.0189 (6)	0.0208 (5)	−0.0004 (5)	0.0104 (5)	0.0029 (5)
C15	0.0165 (5)	0.0216 (6)	0.0192 (5)	0.0010 (5)	0.0109 (4)	0.0013 (5)
C16	0.0174 (5)	0.0192 (6)	0.0269 (6)	0.0016 (5)	0.0140 (5)	0.0027 (5)
C17	0.0163 (5)	0.0188 (6)	0.0202 (5)	−0.0007 (5)	0.0100 (4)	0.0007 (5)
C18	0.0240 (6)	0.0213 (7)	0.0270 (6)	0.0026 (6)	0.0136 (5)	0.0043 (5)
C19	0.0217 (6)	0.0319 (8)	0.0221 (6)	0.0016 (6)	0.0125 (5)	0.0005 (6)
C20	0.0229 (6)	0.0221 (7)	0.0271 (6)	0.0002 (5)	0.0162 (5)	0.0036 (5)
C21	0.0183 (5)	0.0209 (6)	0.0263 (6)	−0.0006 (5)	0.0150 (5)	−0.0014 (5)

### Geometric parameters (Å, °)

**Table:**

O1—C8	1.2288 (15)	C11—C12	1.3819 (18)
O2—N3	1.2364 (13)	C11—H11A	0.9300
O3—N3	1.2297 (14)	C12—C13	1.3910 (17)
N1—C8	1.3668 (15)	C12—H12A	0.9300
N1—N2	1.3764 (15)	C13—C14	1.3892 (16)
N1—H1	0.8906	C14—C15	1.3837 (18)
N2—C9	1.2956 (15)	C14—H14A	0.9300
N3—C13	1.4667 (17)	C15—H15A	0.9300
C1—C2	1.3879 (18)	C16—C17	1.5465 (17)
C1—C6	1.3947 (17)	C16—H16A	0.9700
C1—H1A	0.9300	C16—H16B	0.9700
C2—C3	1.3969 (17)	C17—C18	1.5241 (18)
C2—H2A	0.9300	C17—C19	1.5315 (17)
C3—C4	1.3964 (18)	C17—H17A	0.9800
C3—C16	1.5089 (17)	C18—H18A	0.9600
C4—C5	1.3945 (18)	C18—H18B	0.9600
C4—H4A	0.9300	C18—H18C	0.9600
C5—C6	1.3971 (17)	C19—H19A	0.9600
C5—H5A	0.9300	C19—H19B	0.9600
C6—C7	1.5232 (17)	C19—H19C	0.9600
C7—C8	1.5231 (15)	C20—H20A	0.9600
C7—C20	1.5328 (18)	C20—H20B	0.9600
C7—H7A	0.9800	C20—H20C	0.9600
C9—C10	1.4860 (18)	C21—H21A	0.9600
C9—C21	1.5027 (15)	C21—H21B	0.9600
C10—C15	1.3962 (17)	C21—H21C	0.9600
C10—C11	1.4083 (15)		
			
C8—N1—N2	120.30 (9)	C14—C13—C12	121.66 (12)
C8—N1—H1	116.9	C14—C13—N3	118.64 (11)
N2—N1—H1	122.5	C12—C13—N3	119.70 (10)
C9—N2—N1	116.84 (10)	C15—C14—C13	118.61 (11)
O3—N3—O2	123.84 (12)	C15—C14—H14A	120.7
O3—N3—C13	118.43 (10)	C13—C14—H14A	120.7
O2—N3—C13	117.73 (10)	C14—C15—C10	121.51 (10)
C2—C1—C6	121.19 (11)	C14—C15—H15A	119.2
C2—C1—H1A	119.4	C10—C15—H15A	119.2
C6—C1—H1A	119.4	C3—C16—C17	113.56 (10)
C1—C2—C3	121.25 (12)	C3—C16—H16A	108.9
C1—C2—H2A	119.4	C17—C16—H16A	108.9
C3—C2—H2A	119.4	C3—C16—H16B	108.9
C4—C3—C2	117.64 (11)	C17—C16—H16B	108.9
C4—C3—C16	122.38 (11)	H16A—C16—H16B	107.7
C2—C3—C16	119.96 (12)	C18—C17—C19	110.81 (10)
C5—C4—C3	121.14 (11)	C18—C17—C16	110.33 (10)
C5—C4—H4A	119.4	C19—C17—C16	111.19 (11)
C3—C4—H4A	119.4	C18—C17—H17A	108.1
C4—C5—C6	120.94 (12)	C19—C17—H17A	108.1
C4—C5—H5A	119.5	C16—C17—H17A	108.1
C6—C5—H5A	119.5	C17—C18—H18A	109.5
C1—C6—C5	117.83 (11)	C17—C18—H18B	109.5
C1—C6—C7	120.37 (11)	H18A—C18—H18B	109.5
C5—C6—C7	121.72 (11)	C17—C18—H18C	109.5
C8—C7—C6	110.83 (9)	H18A—C18—H18C	109.5
C8—C7—C20	109.81 (11)	H18B—C18—H18C	109.5
C6—C7—C20	111.38 (10)	C17—C19—H19A	109.5
C8—C7—H7A	108.2	C17—C19—H19B	109.5
C6—C7—H7A	108.2	H19A—C19—H19B	109.5
C20—C7—H7A	108.2	C17—C19—H19C	109.5
O1—C8—N1	119.05 (10)	H19A—C19—H19C	109.5
O1—C8—C7	122.79 (11)	H19B—C19—H19C	109.5
N1—C8—C7	118.16 (11)	C7—C20—H20A	109.5
N2—C9—C10	115.30 (10)	C7—C20—H20B	109.5
N2—C9—C21	123.58 (12)	H20A—C20—H20B	109.5
C10—C9—C21	121.11 (10)	C7—C20—H20C	109.5
C15—C10—C11	118.33 (11)	H20A—C20—H20C	109.5
C15—C10—C9	120.84 (10)	H20B—C20—H20C	109.5
C11—C10—C9	120.81 (11)	C9—C21—H21A	109.5
C12—C11—C10	120.94 (11)	C9—C21—H21B	109.5
C12—C11—H11A	119.5	H21A—C21—H21B	109.5
C10—C11—H11A	119.5	C9—C21—H21C	109.5
C11—C12—C13	118.92 (10)	H21A—C21—H21C	109.5
C11—C12—H12A	120.5	H21B—C21—H21C	109.5
C13—C12—H12A	120.5		
			
C8—N1—N2—C9	178.11 (10)	N2—C9—C10—C15	163.79 (11)
C6—C1—C2—C3	1.07 (17)	C21—C9—C10—C15	−14.89 (17)
C1—C2—C3—C4	−1.20 (17)	N2—C9—C10—C11	−14.87 (16)
C1—C2—C3—C16	177.46 (10)	C21—C9—C10—C11	166.45 (11)
C2—C3—C4—C5	0.48 (17)	C15—C10—C11—C12	−1.73 (17)
C16—C3—C4—C5	−178.13 (10)	C9—C10—C11—C12	176.97 (11)
C3—C4—C5—C6	0.37 (17)	C10—C11—C12—C13	1.28 (18)
C2—C1—C6—C5	−0.19 (16)	C11—C12—C13—C14	−0.03 (18)
C2—C1—C6—C7	176.58 (10)	C11—C12—C13—N3	−179.31 (11)
C4—C5—C6—C1	−0.52 (16)	O3—N3—C13—C14	16.88 (16)
C4—C5—C6—C7	−177.24 (10)	O2—N3—C13—C14	−162.63 (11)
C1—C6—C7—C8	120.04 (12)	O3—N3—C13—C12	−163.82 (11)
C5—C6—C7—C8	−63.31 (14)	O2—N3—C13—C12	16.67 (16)
C1—C6—C7—C20	−117.38 (12)	C12—C13—C14—C15	−0.72 (18)
C5—C6—C7—C20	59.26 (14)	N3—C13—C14—C15	178.56 (10)
N2—N1—C8—O1	−175.54 (10)	C13—C14—C15—C10	0.24 (18)
N2—N1—C8—C7	5.01 (16)	C11—C10—C15—C14	0.95 (17)
C6—C7—C8—O1	96.99 (14)	C9—C10—C15—C14	−177.74 (11)
C20—C7—C8—O1	−26.50 (16)	C4—C3—C16—C17	101.68 (13)
C6—C7—C8—N1	−83.58 (13)	C2—C3—C16—C17	−76.91 (14)
C20—C7—C8—N1	152.94 (11)	C3—C16—C17—C18	166.22 (11)
N1—N2—C9—C10	−178.57 (9)	C3—C16—C17—C19	−70.42 (14)
N1—N2—C9—C21	0.07 (16)		

### Hydrogen-bond geometry (Å, °)

**Table:**

*D*—H···*A*	*D*—H	H···*A*	*D*···*A*	*D*—H···*A*
N1—H1···O1^i^	0.89	2.13	3.0012 (16)	167
C1—H1A···O2^ii^	0.93	2.58	3.4118 (15)	149
C16—H16B···Cg1^iii^	0.97	2.88	3.6113 (16)	133
C18—H18B···Cg2^iv^	0.96	2.99	3.9348 (16)	167
C21—H21C···Cg1^v^	0.96	2.80	3.5600 (16)	137

Symmetry codes: (i) −*x*+1, −*y*+3, −*z*+2; (ii) −*x*+1, *y*+1/2, −*z*+3/2; (iii) −*x*+2, −*y*+2, −*z*+2; (iv) *x*+1, −*y*+1/2, *z*−1/2; (v) −*x*+1, −*y*+2, −*z*+2.
